# A Finnish Bath

**Published:** 1891-03

**Authors:** 


### A FINNISH BATH.

The Baltimore *Sun* has a correspondent who gives the following description of a Finnish bath : The secret of the attraction of the resort lies in its baths. These consist of a large building with small compartments for bathers, and a swimming basin with water from the sea, heated by steam, and ever fresh-running. Each visitor gets a carpeted room, with a lounge to sleep on when done bathing. Stretched on the lounge, one is covered with a heavy layer of mud from the bottom of the sea. The attendant then comes to pinch, beat, press and scrub the body all over for half an hour, until one faints or seems half dead. The next process is to be plunged into the water tub, then brushed with soap, then different kinds of douches for another half hour. Next comes a swim in the great basin, and then a thorough drying by massage. Covered up with blankets, one then goes to sleep like a top, and after—say two hours, the subject of all this process is rubbed and dressed. All of this is done by Finnish women of all ages, dressed in blue skirts, white and red bodices, and with bare feet.

